# MGL Ligand Expression Is Correlated to Lower Survival and Distant Metastasis in Cervical Squamous Cell and Adenosquamous Carcinoma

**DOI:** 10.3389/fonc.2019.00029

**Published:** 2019-01-29

**Authors:** Neha M. Sahasrabudhe, Joost C. van der Horst, Vivian Spaans, Gemma Kenter, Cor de Kroon, Tjalling Bosse, Sandra J. van Vliet, Ekaterina S. Jordanova

**Affiliations:** ^1^Department of Molecular Cell Biology and Immunology, Cancer Center Amsterdam, Amsterdam Infection & Immunity Institute, Amsterdam UMC, Vrije Universiteit Amsterdam, Amsterdam, Netherlands; ^2^Department of Pathology, Leiden University Medical Centre, Leiden, Netherlands; ^3^Department of Obstetrics and Gynecology, Leiden University Medical Centre, Leiden, Netherlands; ^4^Department of Obstetrics and Gynecology, Center for Gynecological Oncology Amsterdam, Amsterdam UMC, Vrije Universiteit Amsterdam, Amsterdam, Netherlands

**Keywords:** MGL, glycosylation, cervical cancer, distant metastasis, histological subtype

## Abstract

Cervical cancer is the fourth most common cancer type in women worldwide and is characterized by a highly immune-suppressive microenvironment. Here, we describe aberrant glycosylation as a factor mediating this immunosuppressive microenvironment. Expression of a specific carbohydrate ligand for the immune-regulatory C-type lectin MGL was correlated to poor disease-specific survival and distant recurrences in squamous cell carcinoma (SCC) and adenosquamous carcinoma (ASC), the most common histological subtypes of cervical cancer. MGL ligand expression was also associated with lymph node metastasis, the absence of CD14^+^ myeloid cells and the presence of CD14^−^CD163^+^ myeloid cells. Indeed, expression of the MGL receptor itself could be detected on CD163^+^ cells, suggesting that MGL^+^ myeloid cells are able to interact locally with MGL ligand^+^ tumor cells. Additionally, MGL ligand expression correlated to the occurrence of *PIK3CA* mutations, the most frequently observed oncogenic alteration in cervical cancer. In conclusion, we present prognostic value for MGL ligand expression in SCC/ASC patients, which further supports an immune evasive role for the C-type lectin MGL in the tumor immune compartment.

## Introduction

Cervical cancer is the fourth most common cancer in women worldwide (1). Histologically cervical cancer is subdivided into squamous cell carcinoma (SCC), adenocarcinoma (AC), and adenosquamous carcinoma (ASC), of which 75–80% are of SCC subtype and 15–20% of AC and ASC combined (2). Almost all SCC, AC, and ASC cervical cancer cases are due to persistent human papilloma virus (HPV) infection and gradually accumulating mutations (3). Although cervical cancer spreads in a predictable stage wise manner, disease recurrence is still a major concern. The primary treatment for stage IB-IIA cervical cancer is either radical surgical removal coupled with adjuvant chemo-radiotherapy or primary chemo-radiotherapy (4). After adjuvant radiotherapy recurrences are only reduced by approximately 50% (5), suggesting that new prognostic markers would be beneficial in predicting disease-free recurrence and therapeutic success.

The development of cancer from the initial stage of HPV infection to the final invasive carcinoma depends on the immune response mounted by the host against HPV. Only 1% of women develop invasive carcinoma following the HPV infection due to an effective cellular immune response against cancerous cells (6). However, in patients having invasive carcinoma, the cancer is characterized by a highly immunosuppressive tumor microenvironment. Increased frequencies of regulatory T cells, and T cells expressing high levels of the immune checkpoint molecules PD-1 and CTLA-4 were observed in lymph nodes with metastasized cervical tumor compared to tumor negative lymph nodes (7). We and others, have reported on various factors mediating tumor immune suppression such as high expression of HLA- E coupled with lower infiltration of CD8^+^ T cells and NK cells (8, 9), reduced HLA class I expression (10), reduced infiltration of M1 type macrophages (11), increased PDL-1 expression on both tumor cells and myeloid cells (7, 12), increased Indoleamine-2,3-dioxygenase (IDO) metabolic activity (13) and reduced major histocompatibility complex class I chain-related molecule A (MICA) expression (14) aiding immune escape by cervical cancer. Also, the different histological subtypes have different genomic and immunological characteristics (9, 14–17).

Aberrant glycosylation is a principal, yet underappreciated, factor mediating tumor immune escape (18). Glycosylation is the most abundant post-translational modification of proteins and lipids. However, during tumor progression an altered glycosylation pattern is observed in tumor cells compared to their non-transformed counterparts (19). Often, elevated expression of tumor-associated glycans is associated with increased aggressiveness, metastasis of the disease and hampered immune recognition of tumors (19). Epithelial cancers, such as cervical cancer, have a highly glycosylated surface and are associated with increased expression of truncated *O*-glycans such as Tn antigen (GalNAcα-Ser/Thr) (20, 21). We have previously demonstrated that Tn antigen expressed on the mucin MUC1 is bound by the glycan-binding receptor macrophage galactose-type lectin (MGL/CLEC10A/CD301) (22). MGL is a C-type lectin receptor expressed specifically in myeloid cells, such immature tolerogenic dendritic cells and macrophages (23). The interaction of MGL with its ligand on effector T cells blocks T cell activation and induces T cell apoptosis (24). Furthermore, in colorectal cancer, high MGL ligand expression in tumors is associated with poor disease-free survival in late stage disease, whereby MGL ligand expression was highly correlated to mutations in the BRAF gene (25). While it is established that MGL ligand binding leads to an immune regulatory response (26), whether MGL binding has a role in mediating immune-evasion and tumor growth in cervical cancer patients has not been determined yet. In the present study we analyzed a cohort of cervical cancer samples for binding to chimeric MGL receptors and determined whether MGL binding was correlated to clinical and immune parameters for disease progression and aggressiveness of the tumor. Our results demonstrate a positive correlation between MGL binding and distant metastasis in cervical (adeno-)squamous cell carcinoma and a reduced recurrence-free survival.

## Materials and Methods

### Subjects and Follow-Up Data

were retrieved from Department of Pathology, Leiden University Medical Center, Leiden, The Netherlands. From 109 patients with cervical carcinoma who underwent radical hysterectomy with bilateral pelvic lymphadenectomy between 1985 and 1999, formalin-fixed, paraffin-embedded tissue blocks were retrieved from the archives. These cases were chosen on the basis of availability of sufficient material. All cases were used according to the Code of Conduct for Proper Secondary Use of Human Tissue, established by the Federation of Dutch Medical Scientific Societies (www.hedera.org) (27). Importantly, all these cases were previously subjected to thorough analyses by additional PAS+/AB staining to determine their exact histological subtype (17). Patients which were treated with radiotherapy and/or chemotherapy prior to surgery were excluded from the sample group. Follow-up data in this database was recorded from the date of primary surgery till September 2016. Follow-up data was recorded prospectively in a de-identified manner and was updated directly from medical records of patients. Follow-up time in months was between primary surgery and: (1) death caused by cervical cancer, while patients who died of a cause unrelated to cervical cancer are considered as censored observations at the date of death (disease-specific survival); and (2) distant metastasis or locoregional recurrence, whichever occurred first, or when both occurred within 30 days both were stated. Death was considered as a censored observation (disease-free survival).

### Immunohistochemistry Using Tissue Microarrays

Tissue microarray (TMA) were created by punching three core biopsies with a diameter of 0.6 mm from the central part of the tumor (28). Core biopsies were transfer to a recipient paraffin block. TMAs were sectioned in 4 μm size, deparaffinized and rehydrated. Endogenous peroxidase was blocked using 0.03% hydrogen peroxide in 70% ethanol for 20 min, followed by incubation with MGL-mouseFc for 2 h at RT (29). Then, slides were washed with PBS and incubated with Goat anti-mouse IgG Fc-PO (Jackson Immunoresresearch Laboratories) for 1 hr. Complexes were visualized using 3,3'-diaminobenzidine tetrahydrochloride (Sigma, USA). Slides were counterstained with Haematoxylin and coverslipped. MGL binding was scored in tumor fields and subsequently grouped in two groups containing negative/weak and positive/strong cases.

### Immunofluorescence

Immunostaining was performed on 4 μm thick formalin fixed paraffin embedded tumor sections using anti-MGL antibodies synthesized in our laboratory (IG6.6, IgG2a) (23) and anti-CD163 antibody (NCL-CD163 LeicaBiosystems, IgG1). Antigen retrieval was performed using Tris–ethylenediaminetetraacetic acid (EDTA) buffer (10 mM Tris plus 1 mM EDTA pH 9.0). Antigen retrieval was followed by overnight incubation at room temperature with anti-MGL and anti-CD163 antibody diluted in 1% w/v bovine serum albumin in PBS. Alexa Fluor labeled Goat-anti-mouseIgG1-A488 (CD163) and Goat-anti-mouseIgG2a-A546 (both from Invitrogen, Life Technologies, Carlsbad, USA) were incubated at room temperature for 1 hour. Slides were mounted using VectaShield mounting medium containing DAPI. Images were obtained using an LSM700 confocal laser scanning microscope containing an LCI Plan-Neofluar 25 × /0.8 Imm Korr DIC M27 objective (Zeiss, Göttingen, Germany).

### Somatic Mutation Genotyping

Previously published data on somatic mutation genotyping using the GynCarta 2.0 mutation panel (Sequenom, Hamburg, Germany) was used (30). This panel analyzes mutations that are most commonly involved in gynecological malignancies, detecting 171 mutations in 13 genes: *BRAF, CDKN2A, CTNNB1, FBXW7, FGFR2, FGFR3, FOXL2, HRAS, KRASNRAS, PIK3CA, PPP2R1A*, and *PTEN*. The mutation spectra of these samples were published previously (17, 30).

### Statistics

To determine association between MGL ligand expression and clinocopathologic characteristics, the chi-squared (χ^2^) test was used. Univariate Cox-regression analysis was performed to assess the association with disease-free survival. Survival curves were estimated by the Kaplan-Meier method. Multivariate Cox-regression analysis was performed to determine whether MGL ligand expression was independently associated with HPV type, FIGO disease staging, histopathological diagnosis, tumor size, infiltration depth, parametrial invasion, vaso-invasion, and lymph node metastasis. These factors were considered as covariates and included in multivariate analyses and if *p* < 0.10 in univariate analysis. To determine the association between MGL ligand expression and oncogenic mutations chi-squared (χ^2^) test was used. Significance tests were two-sided and statistical significance was assumed when *p* < 0.05, corresponding to 95% confidence intervals (CI). Statistical analyses were performed using IBM SPSS Statistics 23. This study is reported according to Reporting recommendations for tumor MARKer prognostic studies (REMARK) (31).

## Results

### MGL Ligand Expression in SCC/ASC Correlates to Lymph Node Metastasis

**Figure 1:**
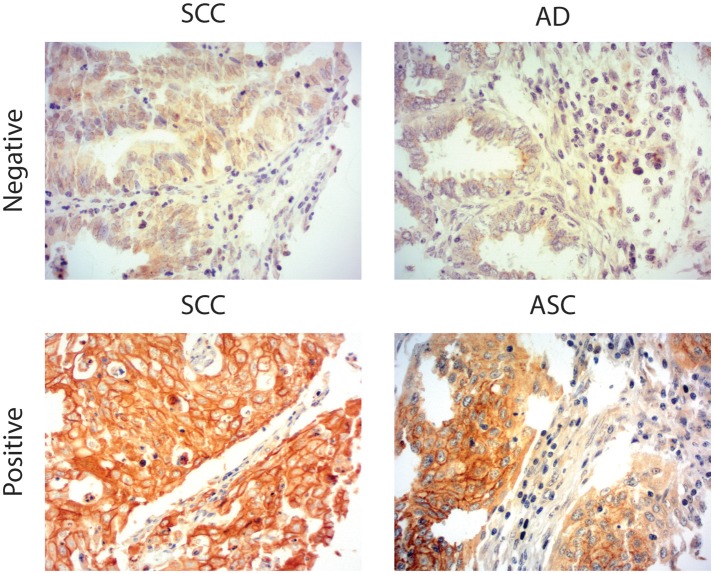
MGL ligand expression in cervical cancer. Representative images of Squamous Cell Carcinoma (SCC), Adenosquamous carcinoma (ASC), and adenocarcinoma (AC) cervical cancer tissues stained with a chimeric MGL-mouseFc protein. MGL ligand expression was labeled positive or negative/weak based on the intensity of MGL-mouseFc binding. Magnification 20x.

Due to the well-defined, highly immunosuppressive tumor microenvironment in cervical cancer, we investigated whether MGL ligand expression is also correlated to different clinical parameters of cervical cancer patients. MGL ligand expression in tumor samples was determined using a chimeric MGL-mouseFc protein, which detects the presence of MGL binding glycans in the tissue (Figure 1). Of the 109 patient samples included in the TMA, MGL staining could be analyzed in 96, others were excluded due to lack of tumor cells or loss of tumor core material. Patients were segregated based on clinicopathological parameters such as HPV subtype, International federation of Gynecology and Obstetrics (FIGO) staging, histopathology, tumor size, tumor infiltration depth, parametrial invasion, vaso-invasion, and lymph node metastasis (Table 1). Among all the parameters analyzed, a significant positive correlation between MGL ligand expression and histopathological subgroups was determined (*p* = 0.02), whereby high MGL ligand expression was observed in 42.4% of the SCC samples and 21.7% of the ASC. In contrast, only 7% of the AC tumors displayed high MGL ligand expression. As MGL ligand expression was mainly restricted to SCC/ASC group of patients, we continued our analysis using these two histological subtypes. We next compared all clinicopathological parameters to the SCC/ASC tumors and observed a significant correlation between MGL ligand expression and a higher frequency of lymph node metastasis (*p* = 0.04).

**Table 1:** Clinicopathologic characteristics of the studied cohort (*n* = 96) in relation to MGL-ligand expression.

	**Total**	**MGL-ligand weak**	**MGL-ligand strong**	
	***N*(%)**	***N*(%)**	***N*(%)**	***P-value***
**HPV TYPE**				
Negative	7 (7.3)	5 (71.4)	2 (28.6)	
16	50 (52.1)	35 (70)	15 (30)	
18	20 (20.8)	14 (70)	6 (30)	
Other	16 (16.7)	10 (62.5)	6 (37.5)	0.1
Unknown	3 (3.1)			
**FIGO**				
IB1	45 (46.9)	29 (64.4)	16 (35.6)	
IB2	31 (32.3)	22 (71)	9 (29)	
IIA	20 (20.8)	14 (70)	6 (30)	0.8
**HISTOPATHOLOGY**				
SCC	59 (61.5)	34 (57.6)	25 (42.4)	
ASC	23 (24)	18 (78.3)	5 (21.7)	
AC	14 (14.6)	13 (92.9)	1 (7.1)	**0.02**
**TUMOR SIZE**				
<40 mm	54 (56.3)	38 (70.4)	16 (29.6)	
≥40 mm	37 (38.5)	22 (59.5)	15 (40.5)	
Unknown	5 (5.2)			0.3
**INFILTRATION DEPTH**				
<15 mm	51 (53.1)	38 (74.5)	13 (25.5)	
≥15 mm	45 (46.9)	27 (60)	18 (40)	0.1
**PARAMETRIAL INVASION**				
Negative	86 (89.6)	60 (69.8)	26 (30.2)	
Positive	10 (10.4)	5 (50)	5 (50)	0.2
**VASOINVASION**				
Negative	45 (46.9)	33 (73.3)	12 (26.7)	
Positive	48 (50)	29 (60.4)	19 (39.6)	0.2
Unknown	3 (3.1)			
**LYMPH NODE METASTASIS**				
Negative	70 (72.9)	51 (72.9)	19 (27.1)	
Positive	26 (27.1)	14 (53.8)	12 (46.2)	0.07 (0.04^*^)
**POSTOPERATIVE RADIOTHERAPY**				
Yes	54 (56.3)	32 (59.3)	22 (40.7)	0.05
No	42 (43.7)	33 (78.6)	9 (21.4)	

*P-value obtained with the Chi-square test*.

* *Statistical values in SCC/ASC patient subgroup. HPV, human papillomavirus; FIGO, international federation of gynecology and Obstetrics stage for cervical carcinoma; SCC, squamous cell carcinoma; ASC, adenosquamous carcinoma; AC, adenocarcinoma. Bold values signify significance*.

### MGL Ligand Expression in SCC/ASC Correlates to Reduced Distant Recurrence-Free Survival

**Figure 2:**
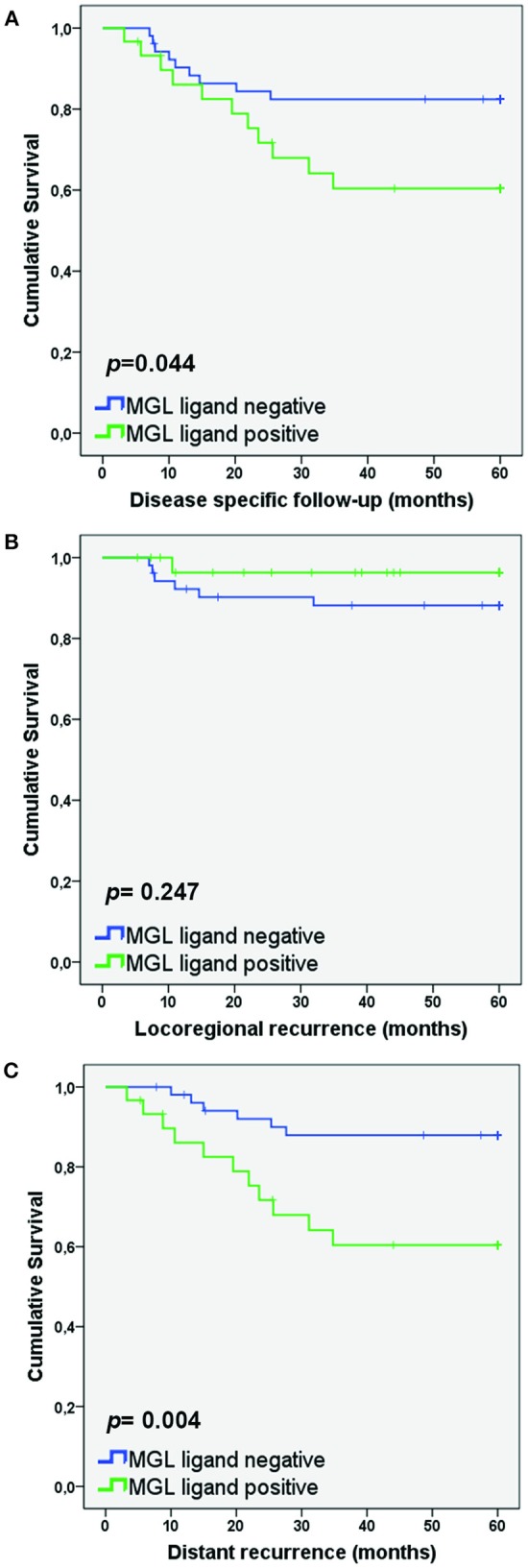
MGL ligand expression correlates to lower survival in patients with distant recurrence. Kaplan Meier survival curves were plotted for SCC/AC patients for Disease-specific survival **(A)**, Locoregional recurrence **(B)**, and Distant recurrence **(C)**. *P-*values determined by Log rank test.

As MGL binding was related to a higher frequency of lymph node metastasis, we also determined whether high MGL binding was associated with a decreased disease-specific survival of patients within the SCC/ASC group. Lower disease-specific survival was found in the high MGL ligand group (*p* = 0.044; Figure 2A). In addition, when we differentiated to recurrence-free survival based on locoregional recurrence and distant recurrence, we observed clear differences between the high and low MGL ligand expressing patient groups. While no statistically significant difference in locoregional recurrence was observed (Figure 2B), MGL ligand expression was significantly correlated to distant recurrences (*p* = 0.004; Figure 2C).

### MGL Receptor Is Expressed on Myeloid Cells in Cervical Cancer Patients

**Figure 3:**
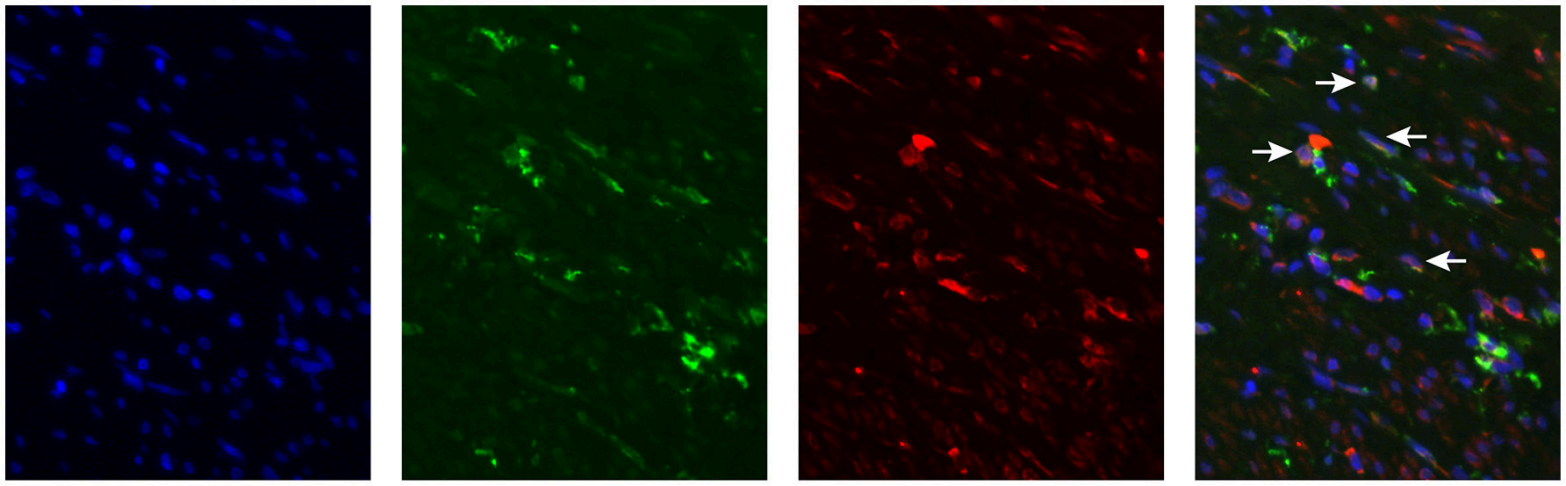
MGL expression in Cervical Squamous cell carcinoma. MGL receptor (red), CD163 (green, myeloid cells) staining in formalin fixed tumor tissue. Nuclei were stained with DAPI (in blue).

We have determined MGL ligand expression in tumor samples from cervical cancer patients. The clinical outcome in patients having MGL ligand expression, however depends equally on expression of MGL receptor in the tumor. Myeloid cell infiltration data, comprising subpopulations expressing CD14, CD33, and CD163 was previously described for this cohort and was integrated in the present study to determine association of MGL ligand expression with myeloid cell subpopulations (11). Using the non-parametric Mann-Whitney test, a positive correlation between CD163^+^CD14^−^ myeloid cells and high MGL ligand expression (*p* = 0.029) was observed, while there were less CD163^−^CD14^+^ cell in MGL ligand positive tumors (*p* = 0.012). We next assessed by double immunofluorescent staining on the same TMA with an anti-MGL and CD163 antibodies, if the MGL receptor was expressed on the same CD163^+^CD14^−^ myeloid cells (Figure 3). Indeed, the MGL receptor was highly expressed in the stroma. Furthermore, MGL co-localization could be observed on a subset of the CD163^+^ tumor-associated myeloid cells (Figure 3).

### MGL Ligand Expression in SCC/ASC Correlates to *PIK3CA* Mutations

We have previously demonstrated that MGL ligand expression is associated with BRAF mutations in colorectal cancer (25). Thus, we sought for possible associations between MGL ligand expression and known somatic mutations in cervical cancer tumors (30). The combined somatic mutation analysis in *BRAF, CDKN2A, CTNNB1, FBXW7, FGFR2, FGFR3, FOXL2, HRAS, KRAS, NRAS, PIK3CA, PPP2R1A*, and *PTEN* genes showed significant correlation to MGL ligand expression (*p* = 0.027), however this correlation was only marginal (Table 2). After individual analysis of mutations in *BRAF, CDKN2A, CTNNB1, FBXW7, FGFR2, FGFR3, FOXL2, HRAS, KRASNRAS, PIK3CA, PPP2R1A*, and *PTEN* genes with respect to MGL ligand expression, we only observed a significant correlation only to E542K and E545K mutations in PI3K (*p* = 0.006; Table 2).

**Table 2:** MGL ligand expression in relation to mutational status.

	**Total**	**Weak MGL-ligand expressions**	**Strong MGL-ligand expression**	
	***N*^**a**^(%)**	***N*(%)**	***N*(%)**	***P-value***
**SOMATIC MUTATIONS**				
No	56 (68.3)	40 (71.4)	16 (28.6)	
Yes	26 (31.7)	12 (46.1)	14 (53.8)	0.027
**PI3K MUTATIONS**				
No	63 (76.8)	45 (71.4)	18 (28.6)	
Yes	19 (23.2)	7 (36.8)	12 (63.2)	0.006

a *Total number of cases = 82*.

*P-value obtained with the Chi-square test*.

## Discussion

Aberrant glycosylation in the tumor microenvironment is recognized as one of the principal factors mediating aggressive tumor growth (32). Certain glycan structures have a pivotal role in mediating immune suppression in tumor microenvironment. Binding of tumor associated glycan ligands such as the Tn antigen to the MGL receptor is one such mechanism by which tumors succeed in immune evasion accompanied by the aggressive tumor phenotype and lower recurrence-free survival (19, 24, 25). Here we determined that in (adeno-)squamous cervical cancer, high levels of MGL ligand expression correlated to lymph node metastasis and reduced distant recurrence-free survival. Thus, similar to colorectal cancer, also in cervical cancer MGL ligand expression was associated with a more aggressive tumor phenotype (25). Concomitant with the high levels of MGL ligands, MGL receptor expression was observed in myeloid cells in tumor stroma, more specifically in CD163^+^ myeloid cells. Furthermore, the association between MGL ligand expression and oncogenic *PIK3CA* mutations suggest a causative role of *PIK3CA* mutation in MGL ligand expression in cervical cancer. Thus, here we elaborated on the role of MGL ligand expression and aberrant glycosylation in cervical cancer progression.

In our analysis, MGL ligand expression was mainly observed in SCC/ASC and not in the AC histological subtype. Among the histological subtypes of cervical cancer, SCC is the most predominant histological subtype followed by ASC/AC accounting for about 10–15% of total patients (33, 34). Apart from the histological differences between the subgroups, AC differs from both SCC and ASC with respect to metastasis, pattern of tumor growth, therapeutic regime as well as oncogenic mutations (17, 35–37). Analyses from our previous studies have demonstrated a significant difference between AC and SCC/ASC with respect to frequencies of an immunosuppressive microenvironment, where AC show improved survival with high immunosuppressive environments, while SCC/ASC show the opposite (9, 14, 16). The non-classical HLA molecule HLA-E is weakly expressed in healthy cervical epithelium while its expression increases with progression to higher stages of cancer (8, 38). HLA-E is proven to be a mediator for immune escape in virally infected cells and tumor cells (8, 39, 40). Previously, we have shown that HLA-E expression is significantly higher in the AC subgroup than in SCC/ASC patients and high HLA-E expression was correlated to improved disease-specific and recurrence-free survival (9). Complete loss of classical HLA class I molecules is observed more on SCC than in AC, again a characteristic of a highly immunosuppressive microenvironment (10, 41). In the same line of reasoning, a higher frequency of Tregs was correlated to better survival in AC (16) but poorer survival in SCC (42, 43). Thus, an immunosuppressive microenvironment specifically results in poor survival in SCC patients, in contrast to patients with AC. This contrasting role of the immune microenvironment was also observed in the present study in relation to MGL ligand expression. We observed MGL ligand expression mainly in SCC/ASC groups, while minimal expression was observed in AC. MGL ligand expression in SCC/ASC also correlated to lymph node metastasis, reduced disease-specific survival and reduced distant recurrence-free survival. Thus, we propose that expression of MGL ligands in SSC/ASC is one of the factors mediating immune suppression which is correlated to poor survival, while the same mechanism might not apply to patients in the AC subgroup. These results further corroborate the differences between histological subtypes of cervical cancer and the need for opposing prognostic markers and therapeutic strategies.

Persistent carcinogenic HPV infection is the causative agent in 95% of cervical cancer patients. Following HPV infection, disease progression is frequently accompanied with genomic instability and accumulation of somatic mutations (15). The mutational profile of cervical cancers is a factor determining disease outcome as well as the histological subtype differentiation (15, 17). Among the somatic mutations observed, activating mutations in the *PIK3CA* gene, encoding the catalytic subunit of PI3K, are the most frequently observed mutations accounting for 20–36% of cervical cancer tumors (17, 37, 44–46). The PI3K-AKT-mTOR pathway controls several aspects of tumor growth such as avoiding cell death, metabolism, metastasis and angiogenesis (47). PI3K activation has been implicated in glycosylation pathways before. In bladder cancer, the PI3K-AKT-mTOR pathway was associated with sialyl-Tn antigen, whereby inhibition of mTOR led to reduced expression of this sialyl-Tn antigen (48). Additionally, expression of β1,6GlcNAc-branched N-glycans on β4 integrin is regulated by the PI3K pathway. Galectin-3 binding to β1,6GlcNAc-branched N-glycans on β4 integrin mediates tumor growth and progression (49). Here, we observed a positive correlation between PI3K mutations and MGL ligand expression. With respect to tumor-associated glycosylation, we have determined that in colorectal cancer, BRAF mutations drive the expression of MGL ligands such as Tn antigen (25, 50). Intriguingly, mutations in the MAPK pathway also constitutively activate the PI3K pathway (47), suggesting that the PI3K-AKT-mTOR pathway could be responsible for enhancing MGL ligand expression. However, further investigation is needed for confirm this causative mechanistic link.

Myeloid cells in the tumor microenvironment are critical factors determining the aggressive nature of a tumor (51). Frequency and polarization of tumor-infiltrating myeloid cells are correlated to disease progression and disease-free survival. CD14^+^CD33^−^CD163^−^ myeloid cells are correlated to high frequencies of cytotoxic T cells and higher influx of both these cell types together is associated with improved disease-specific survival (11). Interestingly, here we found an association of high numbers of this CD14^+^ subpopulation with low MGL ligand expression. In addition, we observed a positive correlation between CD14^−^CD163^+^ myeloid cells and MGL ligand expression. Moreover, MGL receptor expression could be visualized on CD163^+^ cells. The MGL receptor is known to be expressed on myeloid cells such as macrophages and dendritic cells, where engagement of MGL ligands with MGL has been described to induce immune tolerance (26, 52). CD14^−^CD163^+^ myeloid cells, which we observed to be correlated to high MGL ligand expression have previously been correlated to a high frequency of CD3^+^CD8^−^Foxp3^+^ regulatory T cells (11). Thus, our results are in coherence with the known immune-suppressive mechanisms of MGL triggering.

The here described cohort has several limitations; there is a limited number of adenocarcinoma patients and more than half of the patients included received post-surgical treatment radiotherapy. Although this did not have an effect on the role of MGL expression in SCC/ASC survival, a confirmation of our findings in an independent cohort of early stage cervical cancer patients is needed. Moreover, we were not able to study MGL expression in advanced disease as these patients receive chemotherapy and no surgical treatment.

Cervical cancer progression follows a predictable pattern starting with a persistent oncogenic HPV infection, followed by uncontrolled proliferation, eventually leading to metastasis to local lymph nodes and finally distant metastasis (53). Five year survival rates of patients with distant metastasis is drastically reduced to 16.5% compared to localized cervical cancer at 91.5% (54). Novel therapeutic regimes to tackle this dismal survival rate in late stage patients involve immunotherapy against the cancer (55). Aberrant glycosylation is increasingly recognized as factor mediating immune escape in the tumor microenvironment and blocking these immunosuppressive glycans is being explored as a new therapeutic option (56). In this article we have determined that MGL ligand expression is linked to both lymph node and distant metastasis in squamous cervical tumors. Additionally, MGL ligand expression is correlated to *PIK3CA* mutations, the most frequent oncogenic mutation in cervical cancer. Thus, MGL ligand expression may be utilized as a new prognostic and therapy outcome marker. Additionally, blocking of MGL could present as a novel therapeutic option for improved immune eradication of (adeno-) squamous cervical tumors.

## Author Contributions

NS assembled data and wrote the article. JvdH performed data confirmation. VS, GK, CdK, and TB were involved in collecting the data. SvV and EJ did study design and final review of manuscript. All authors have approved the final manuscript.

### Conflict of Interest Statement

The authors declare that the research was conducted in the absence of any commercial or financial relationships that could be construed as a potential conflict of interest.
